# Dermoscopy case: Rupioid psoriasis

**DOI:** 10.1016/j.jdcr.2025.03.004

**Published:** 2025-03-25

**Authors:** Mahesh Mathur, Sandhya Regmi, Sumit Paudel, Nabita Bhattarai, Sambidha Karki, Himanshu Pathak

**Affiliations:** Department of Dermatology, College of Medical Sciences, Bharatpur, Nepal

**Keywords:** dermoscopy, general dermatology, rupioid psoriasis

## Clinical presentation

**Fig 1:**
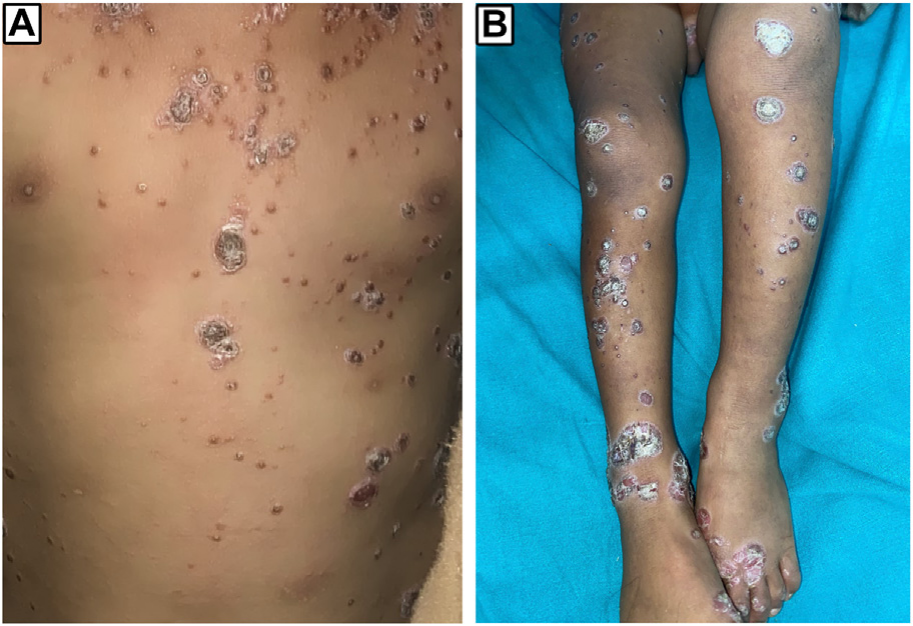
Multiple erythematous, thick, hyperkeratotic plaques with firmly adherent scales are present over the anterior trunk (**A**) and bilateral lower extremities (**B**).

A 2-year-old boy presented with sharply demarcated, erythematous, thick, hyperkeratotic plaques with firmly adherent scales resembling oyster shells over the trunk and bilateral upper and lower extremities for 1 month (Fig 1, *A* and *B*). There is no history of any chronic illness or regular intake of any medication. The HIV and syphilis serology were negative.

## Dermoscopic appearance

**Fig 2:**
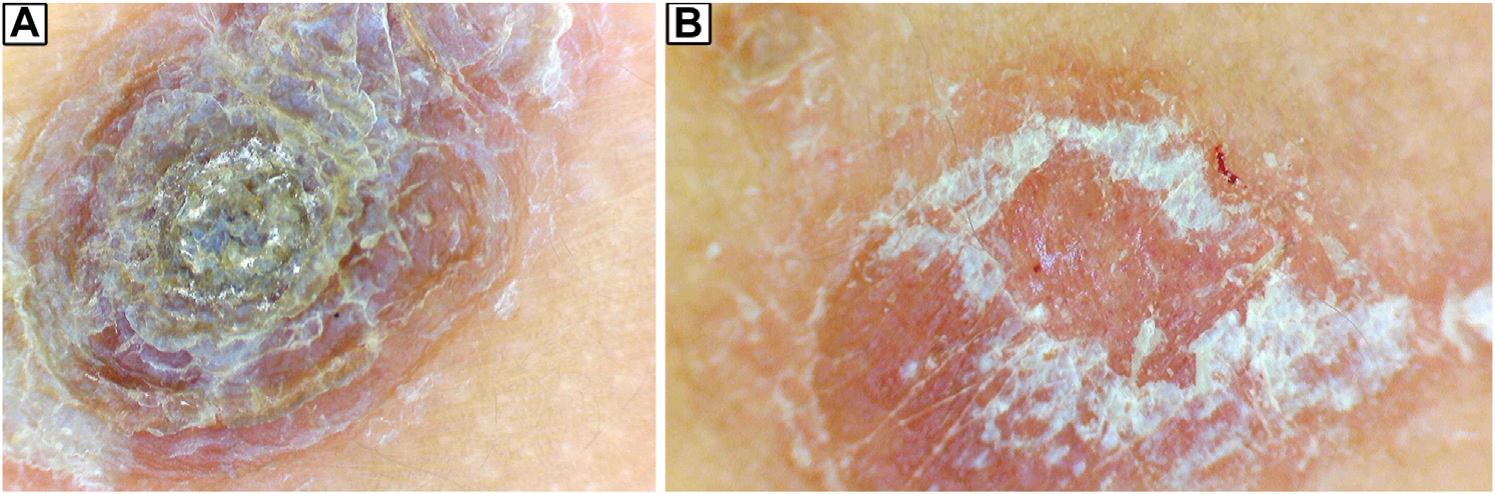
Dermoscopy revealed thick oyster shell-like scales obscuring the projection of the vascular pattern of psoriasis (**A**) along with regularly distributed dotted vessels in a *reddish-pink* background and *white* scales (**B**).

Dermoscopy revealed thick, oyster shell-like scales obscuring the projection of the vascular pattern of psoriasis (Fig 2, *A*) along with regularly distributed dotted vessels in a reddish-pink background and white scales (Fig 2, *B*).

## Histologic diagnosis

**Fig 3:**
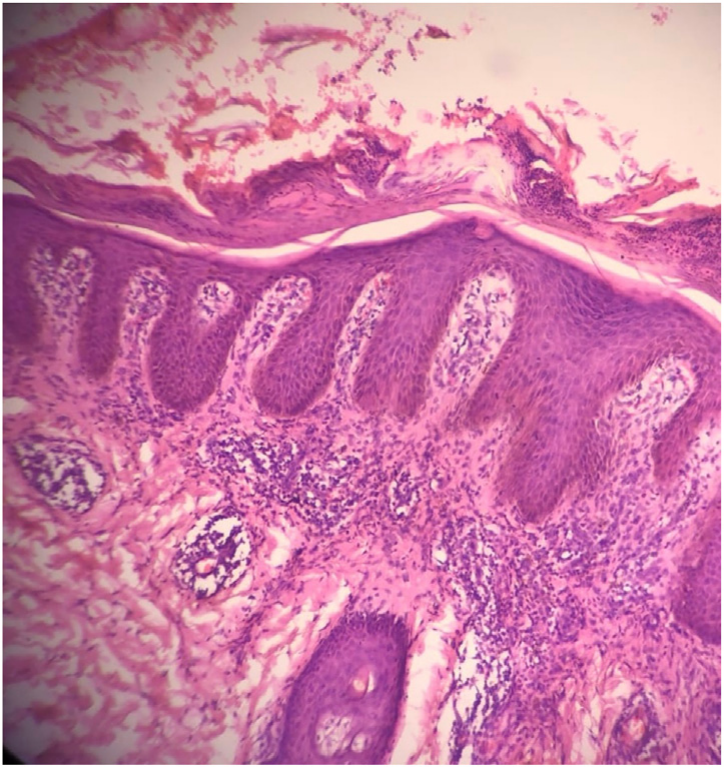
Hematoxylin and eosin staining (10×) showed hyperkeratosis, parakeratosis, acanthosis, thin suprapapillary plates, an absent granular layer, and prominent papillary dermal edema.

Histopathologic examination demonstrated hyperkeratosis with parakeratosis, acanthosis, thin suprapapillary plates, an absent granular layer, and prominent papillary dermal edema leading to the diagnosis of rupioid psoriasis (Fig 3).

**Key message**

The term “rupioid” is used to describe a well-defined, grossly cone-shaped, circular, thick keratotic lesion resembling the limpet shell or oyster shell, which can be observed in psoriasis, scabies, secondary syphilis, disseminated histoplasmosis, sarcoidosis, or acrokeratosis paraneoplastica.^1^ Rupioid psoriasis is a rare subtype of psoriasis that can occur in isolation, in association with HIV, or triggered by medications, including lithium, malarials, and β-blockers.^1^^,^^2^ Histopathologically, rupioid psoriasis is characterized by hyperkeratosis with confluent parakeratosis, Munro microabscesses, diminished granular layer, regular acanthosis, and dilated vessels within the dermis.^1^^,^^2^

Dermoscopy can aid in rendering a timely diagnosis. Typical dermoscopic features of psoriasis are regularly distributed dotted vessels in a reddish-pink background and white scales, while in rupioid psoriasis, thick oyster-shell-like scales obscuring the vascular pattern are also seen.^1^^,^^2^

## Conflicts of interest

None disclosed.
